# Comparison of adult HIV prevalence from national population-based surveys and antenatal clinic surveillance in countries with generalised epidemics: implications for calibrating surveillance data

**DOI:** 10.1136/sti.2008.030452

**Published:** 2008-07-22

**Authors:** E Gouws, V Mishra, T B Fowler

**Affiliations:** 1 Epidemiology and Analysis Division; Evidence, Monitoring and Policy Department; Joint United Nations Programme on HIV/AIDS (UNAIDS), Geneva, Switzerland; 2 Demographic and Health Research Division, Macro International Inc, Calverton, Maryland, USA; 3 US Census Bureau, Washington, USA

## Abstract

**Background:**

Estimates of the impact of HIV in countries with generalised epidemics are generally based on antenatal clinic surveillance data collected over time. In an attempt to obtain geographically more representative estimates of HIV prevalence, many countries are now also conducting national population-based surveys in which HIV testing is included. We compare adult HIV prevalence estimates from antenatal clinic surveillance to those from national population-based surveys to assess the implications for calibrating surveillance data.

**Methods:**

HIV prevalence estimates derived from fitting prevalence curves to antenatal clinic surveillance data are statistically compared to prevalence from national population-based surveys using data from 26 countries with generalised epidemics for the year in which the survey was conducted. Appropriate transformations are applied to inform the correction factors needed to adjust prevalence in countries where population-based surveys have not been conducted.

**Results:**

HIV prevalence derived from antenatal clinic surveillance data generally overestimate population-based survey prevalence by about 20% (95% confidence interval: 10% to 30%) in both urban and rural areas.

**Conclusions:**

In countries where national population-based HIV surveys have been conducted, survey estimates of HIV prevalence (adjusted for potential survey biases as appropriate) can be used directly to calibrate antenatal clinic surveillance data. In countries where national HIV surveys have not been conducted, HIV prevalence derived from antenatal clinic surveillance data should be multiplied by about 0.8 to adjust for overestimation.

Facility-based sentinel surveillance of HIV has been recommended for monitoring the HIV epidemic since the mid-1980s, mainly because of easy access to people attending public health facilities.^1^ In countries with generalised HIV epidemics, defined by UNAIDS and the World Health Organization as countries where adult prevalence is firmly established in the general population and transmission occurs mostly through heterosexual sex,^2^ annual HIV surveillance among pregnant women attending public-sector antenatal clinics has over time become the primary source of data on the spread of HIV.^3^

Concerns about the limitations associated with antenatal clinic surveillance have in recent years led to an increase in the number of countries conducting large national population-based surveys in which HIV testing has been included. These surveys are geographically more representative than antenatal clinic surveillance and include samples from urban and rural areas, men and women and different age groups.

Both sentinel surveillance and population-based surveys have strengths and weaknesses, but taken together provide complementary information and can provide a clearer picture of both overall trends and geographical distribution of HIV in a country.^4^ While national population-based surveys provide better coverage of the general population, sentinel surveillance provide important information on prevalence trends over time.

UNAIDS/WHO estimates of national adult HIV prevalence and the demographic impact of HIV in countries with generalised epidemics have been based on prevalence data collected over time from pregnant women attending antenatal clinics.^5^ ^6^ Because of differences in prevalence between urban and rural areas, country-specific prevalence is often estimated separately for urban and rural areas, and then combined to obtain a national, weighted estimate of adult prevalence.^7^ Historically, the prevalence curve for non-urban areas was adjusted downward by 20% because surveillance systems often did not cover rural areas well, and it was assumed that HIV prevalence was lower in those areas that were excluded from surveillance.^5^ In more recent years, in countries where national population-based surveys have been conducted, prevalence curves fitted to antenatal clinic surveillance data have been calibrated so that the fitted prevalence agrees with the reported population-based survey prevalence estimate for the year in which the survey was conducted.^8^

In addition to using population-based survey estimates of adult HIV prevalence to calibrate adult prevalence curves fitted to antenatal clinic data in countries where such population-based surveys have been conducted, a comparison of these estimates can also be used to inform the adjustment factor needed to correct for potential surveillance bias in countries where national population-based surveys have not been conducted. In this paper we compare adult HIV prevalence estimates from national population-based surveys to those derived from antenatal clinic surveillance to assess the implications for calibrating surveillance data.

## METHODS

### Antenatal clinic surveillance

HIV surveillance has been carried out among women attending antenatal clinics in more than 115 countries worldwide.^3^ In 2006, it was estimated that more than 600 sites in sub-Saharan Africa have been included in surveillance efforts on a regular basis.^9^ These sentinel surveys are usually conducted annually or bi-annually around the same time of the year and involve anonymous, unlinked sampling of blood from pregnant women attending selected antenatal clinics in the public health sector. The main strength of antenatal clinic surveillance is that it provides ready and easy access to a cross-section of sexually active pregnant women from the general population, and it can be used to assess trends in the epidemic over time. In generalised epidemics, HIV prevalence among pregnant women has been considered a good approximation of prevalence among sexually active men and women aged 15–49 years.^10^ In several countries geographical coverage of HIV surveillance systems has expanded over time according to the needs and available resources, often improving the representativeness of samples.

A major limitation of sentinel surveillance systems, however, is limited geographical coverage because sampling is often not representative of smaller and more remote areas in a country.^4^ Antenatal clinic surveillance provides information on women of reproductive age, while estimates for men are based on assumptions about the ratio of male-to-female prevalence derived from community surveys. Furthermore, women attending public antenatal clinics might not be representative of all pregnant women in a country, because some women may receive antenatal care in private clinics, some at home through outreach services and others may receive no professional care. The World Health Organization in 2005 estimated that antenatal care coverage (with at least one antenatal care contact during pregnancy, including care received at private clinics and through outreach services) varied from 27% to 99% (median across 34 countries of 85%) in sub-Saharan Africa.^11^ While sentinel surveillance provides the best source of data on prevalence trends in a country, analysis of these data should be done carefully because of changing geographical surveillance coverage over time, and because the composition of women attending antenatal clinics may change over time as fertility rates change. Furthermore, the quality of surveillance may vary across regions and over time depending on available resources.

### National population-based surveys

Since 2000, more than 25 countries in sub-Saharan Africa have conducted national population-based household surveys, including demographic and health surveys (DHS) and AIDS indicator surveys (AIS), in which biological specimens for HIV testing have been collected at a national level (www.measuredhs.com). These surveys include large nationally representative probability samples of adult men and women and generally use dried blood spots for collecting specimens for HIV testing. The main strength of population-based surveys is that they can provide estimates of HIV prevalence in the general population as well as for different subgroups in different geographical locations, for women and men, and for people in different age groups.^4^ Another major strength is that HIV serostatus can be linked to social, behavioural and other biomedical information, providing the opportunity to study the dynamics of the epidemic in more detail.

The main limitation of population-based surveys is potential bias arising from refusal of study subjects to participate in the survey or to have blood taken for HIV testing, as well as absence of study subjects from the household at the time of the survey. Furthermore, sampling from households in population-based surveys may not adequately represent high-risk non-household population groups and mobile populations. In countries with concentrated or low-level epidemics, population-based surveys might therefore underestimate HIV prevalence and household surveys may be less useful in determining national prevalence in these settings.^4^ ^12^ A further limitation is that population-based household surveys can generally not be conducted frequently because they are logistically difficult and expensive to carry out, and have therefore not been used to assess epidemic trends over time.

### Using population-based surveys as “gold standard” for estimating adult HIV prevalence

The reliability of HIV prevalence estimates obtained from population-based household surveys depends on sound survey and sampling methodology, including taking representative samples of the adult population in relevant subgroups, ensuring high quality of data and specimen collection and employing sound laboratory methods for HIV testing while maintaining high ethical standards. A major challenge in population-based surveys is to minimise the level of non-response, either due to refusal to participate or due to absence of household members at the time of the survey. Mishra and colleagues^13^ studied the impact of non-response on HIV prevalence in national population-based surveys in 14 countries and showed that non-response did not significantly bias HIV estimates. Even though the HIV prevalence among the non-responders in the surveys was predicted to be higher than those who were tested for HIV, the overall effects of non-response on observed prevalence were small and insignificant. In another analysis of data from population-based surveys conducted in 20 countries with generalised epidemics in sub-Saharan Africa, Calleja *et al*^9^ confirmed that for non-response to have a significant effect on observed HIV prevalence, either the non-response rate or the risk of HIV prevalence among non-responders relative to survey respondents, or both, has to be substantial.

A further concern with household surveys is that they exclude mobile populations and people not living in households (for example, people living in hostels, military or police barracks, refugee camps, brothels and prisons) among whom HIV prevalence is likely to be higher than among people living in households. Excluding these groups could lead to an underestimate of national prevalence. Mishra *et al*,^13^ based on an evaluation of potential bias due to exclusion of non-household groups in population-based surveys, conclude that the effect of excluding non-household population groups on the national estimates of HIV prevalence obtained from a household sample is likely to be small in countries with generalised epidemics. Moreover, given the small proportion of the non-household population relative to the total population, even in countries with concentrated epidemics, HIV prevalence among the non-household population needs to be orders of magnitude higher than among the household population for exclusion of non-household groups to have a significant effect on national prevalence based on the household population.

The response rates in the population-based surveys used in this paper were generally high, with the exception of South Africa and Botswana where the national response rate was below 70%,^14^ ^15^ and the Lilongwe district in Malawi where the response rate was 38%.^16^ Urban and rural comparisons could, however, not be made between the survey and antenatal clinic-based prevalence estimates for Botswana and South Africa, and these two countries were excluded from the analysis. In Malawi, the HIV prevalence for the Lilongwe district as well as for the country as a whole was adjusted for the effect of non-response.^16^ In all other countries used in this analysis, the survey response rate was greater than 70% among men and women combined,^13^ methodology and laboratory procedures were sound and it was assumed that the population-based surveys provided high quality, reliable and representative national estimates of HIV prevalence.

### Fitting a curve to national antenatal clinic data over time

The Estimation and Projection Package (EPP) version 2007^17^ was used to fit prevalence curves to antenatal clinic surveillance data collected over time (1985-2007) by national AIDS councils or ministries of health (epidemiological fact sheets, available at www.who.int). Using a maximum likelihood procedure, the EPP model fits curves to HIV epidemics by varying four parameters^7^ ^8^; the rate of growth of the epidemic (*r*); the start year of the epidemic (*t*_0_); the fraction of the population considered to be at risk of infection at the start of the epidemic (*f*_0_); and a behavioural response parameter which determines the final epidemic prevalence (*φ*).

Surveillance systems in most countries have expanded over time, often into areas or settings with lower prevalence than those initially and deliberately selected because of known HIV cases in such areas. In order to deal with such expanding surveillance systems, EPP has incorporated a level fitting procedure, which is described in detail elsewhere.^8^ The procedure is based on the approximation that, while there are variations in absolute prevalence levels from one site to the next, the overall trend of rising and falling prevalence is the same throughout the region being modelled—that is, that all sites in a particular region are assumed to follow similar prevalence patterns over time.

### Comparison of antenatal clinic and population-based survey estimates of HIV prevalence

For countries with generalised epidemics, published reports of national population-based surveys since 2000, including the demographic and health surveys (DHS) and AIDS indicator surveys (AIS) (available at www.measuredhs.com) that included HIV prevalence measurement, were reviewed. In addition, three preliminary reports of DHS conducted in 2006–7 in Central African Republic,^18^ Liberia^19^ and Swaziland^20^ were reviewed. HIV prevalence among adults (aged 15–49 years) was recorded for urban and rural areas separately. In national surveys where the level of non-response could potentially bias HIV estimates, specifically in Ethiopia^21^ and Malawi,^16^ HIV prevalence among survey participants was adjusted according to the predicted prevalence among the non-responders, and the adjusted prevalence estimates were used in the analysis.

For those countries in which national population-based surveys have been conducted, an independent estimate of adult HIV prevalence was derived from fitting a curve using EPP to available antenatal clinic prevalence data over time. For comparison purposes the unadjusted, fitted estimate of HIV prevalence was recorded for urban and rural areas for the same year in which the survey was conducted. We used the fitted prevalence estimates rather than the median prevalence across surveillance sites because the fitted curve takes account of variation in estimates and is based on an average over time rather than on the prevalence recorded for one year only. In addition, some countries do not conduct antenatal clinic surveillance every year and for several countries antenatal clinic surveillance was not done in the same year as the survey. However, with EPP a fitted estimate could be obtained for each year, including the year in which the survey was conducted.

The two sets of prevalence estimates were compared for 26 countries with generalised epidemics in which national surveys have been conducted and for which urban and rural estimates were available, assuming the prevalence from population-based surveys to be the “gold standard”. The ratio of population-based survey to antenatal clinic-based prevalence was calculated for each of the 26 countries for the year in which the survey was conducted. The medians, means and standard errors of the sets of urban and rural ratios were then calculated. To standardise the variance in the data, we applied a probit transformation^22^ to population-based and antenatal clinic derived prevalence estimates, of the form Y′  =  Φ^-1^(*p*), where Y′ is the probit-transformed value, *p* is the prevalence, and Φ^-1^ is the inverse of the cumulative standard normal distribution. We then calculated the median of the differences in probit-transformed prevalences. These median values (for the unadjusted survey: antenatal clinic prevalence ratio and for the probit-transformed prevalence difference across countries) were then used to inform the adjustment factors needed to correct antenatal clinic-based prevalence estimates in countries.

The two correction factors (based on the median ratio or the probit-transformed median difference) were then applied to the antenatal clinic-based estimates of prevalence for each country to determine how well the adjusted estimates agree with the “gold standard” population-based survey prevalence.

## RESULTS

Table 1 shows the comparison between HIV prevalence in urban and rural areas estimated from population-based surveys and derived from antenatal clinic data for 26 countries with generalised epidemics in which population surveys have been conducted, for the year in which the survey was conducted. The majority of countries (24) were from sub-Saharan Africa, with two additional countries (Haiti and the Dominican Republic) from the Caribbean. For three countries in southern Africa, estimates of adult (age 15–49 years) HIV prevalence were not available for urban and rural areas, either from the population-based survey (Botswana) or from antenatal clinic surveillance over time (South Africa and Swaziland). In Liberia, Sierra Leone, Equatorial Guinea and the Dominican Republic, antenatal clinic surveillance covered urban areas, but not rural areas.

**Table 1:** HIV prevalence estimates from population-based surveys and from EPP curves fitted to antenatal clinic (ANC) data

Region	Country	Year of survey	Type of survey	National adult HIV prevalence	Urban				Rural			
					Survey HIV prev	ANC based HIV prev	Ratio survey: ANC	Probit diff	Survey HIV prev	ANC based HIV prev	Ratio survey: ANC	Probit diff
Southern Africa	Lesotho	2004	DHS	23.5	29.1	32.3	0.901	−0.091	21.9	24.3	0.901	−0.079
Southern Africa	Malawi	2004	DHS	12.7	18.3	18.4	0.995	−0.004	11.3	12.8	0.883	−0.075
Southern Africa	Zambia	2002	DHS	15.6	23.1	26.3	0.878	−0.101	10.8	12.7	0.850	−0.097
Southern Africa	Zimbabwe	2005	DHS	18.1	18.9	21.9	0.863	−0.106	17.6	18.8	0.936	−0.045
East Africa	Burundi	2002	Household survey^23^	3.6	9.4	11.8	0.797	−0.131	2.5	2.5	1.000	0.000
East Africa	Ethiopia	2005	DHS	1.6	5.6	10.5	0.533	−0.336	0.7	1.9	0.368	−0.382
East Africa	Kenya	2003	DHS	6.7	10.0	11.0	0.909	−0.055	5.6	8.6	0.651	−0.224
East Africa	Rwanda	2004	DHS	3.0	7.3	5.9	1.237	0.109	2.2	2.7	0.815	−0.087
East Africa	Tanzania	2004	AIS	7.0	10.9	11.2	0.973	−0.016	5.3	5.5	0.964	−0.018
East Africa	Uganda	2004	Sero-behavioural survey^24^	6.4	10.1	9.2	1.098	0.053	5.7	4.3	1.326	0.136
West/Central Africa	Benin	2006	DHS	1.2	1.7	1.9	0.895	−0.045	0.9	1.2	0.750	−0.109
West/Central Africa	Burkina Faso	2003	DHS	1.8	3.6	3.9	0.923	−0.037	1.3	1.6	0.813	−0.082
West/Central Africa	Cameroon	2004	DHS	5.5	6.7	8.3	0.807	−0.113	4.0	6.8	0.588	−0.260
West/Central Africa	Central African Republic	2006	DHS	6.2	8.3	13.4	0.619	−0.277	4.7	15.4	0.305	−0.655
West/Central Africa	Chad	2005	Household survey^25^	3.3	7.0	4.9	1.429	0.179	2.3	2.8	0.821	−0.084
West/Central Africa	Cote d’Ivoire	2005	AIS	4.7	5.4	6.7	0.806	−0.109	4.1	4.6	0.891	−0.054
West/Central Africa	Equatorial Guinea	2004	Household survey^26^	3.2	3.3	3.1	1.065	0.028	3.1			
West/Central Africa	Ghana	2003	DHS	2.2	2.3	3.5	0.657	−0.183	2.0	3.3	0.606	−0.215
West/Central Africa	Guinea	2005	DHS	1.5	2.4	5.0	0.485	−0.328	1.0	3.2	0.313	−0.474
West/Central Africa	Liberia	2007	DHS	1.5	2.5	4.8	0.521	−0.295	0.8			
West/Central Africa	Mali	2001	DHS	1.7	2.2	3.6	0.611	−0.215	1.5	2.6	0.577	−0.227
West/Central Africa	Niger	2002	Household survey^27^	0.9	2.1	2.5	0.840	−0.074	0.6	1.3	0.462	−0.286
West/Central Africa	Senegal	2005	DHS	0.7	0.7	1.5	0.467	−0.287	0.7	1.4	0.500	−0.260
West/Central Africa	Sierra Leone	2005	Household survey^28^	1.5	2.1	4.2	0.500	−0.306	1.3			
Caribbean	Dominican Republic	2002	DHS	1.0	0.9	2.0	0.462	−0.301	1.2			
Caribbean	Haiti	2005	DHS	2.2	2.3	5.1	0.451	−0.360	2.0	2.3	0.870	−0.058
	Median						0.824	−0.107			0.814	−0.092
	Mean						0.797	−0.131			0.736	−0.165
	SEM						0.050	0.030			0.054	0.038
	SD						0.257	0.148			0.252	0.175

The median ratio of population-based survey to antenatal clinic-derived prevalence estimates across countries was 0.82 (interquartile range: 0.55–0.97) for urban and 0.81 (interquartile range: 0.58–0.89) for rural areas. Corresponding mean ratios were 0.80 (95% CI: 0.69 to 0.90) and 0.74 (95% CI: 0.62 to 0.85), respectively.

**Figure 1:**
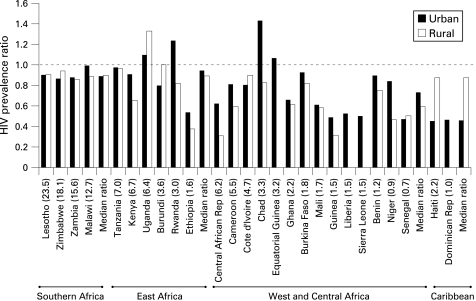
Ratio of population-based adult HIV prevalence to prevalence derived from antenatal clinic data for the year in which the survey was conducted, by country and region. The national adult HIV prevalence from population-based surveys is given in parenthesis for each country.

For a majority of the countries, the prevalence ratio in both urban and rural areas was smaller than 1, indicating that estimated adult HIV prevalence derived from antenatal clinic surveillance almost always overestimates adult prevalence in countries with generalised epidemics (fig 1). The exceptions were Rwanda, Chad and Equatorial Guinea, where urban prevalence from the population-based surveys was higher than the corresponding prevalence derived from antenatal clinic surveillance, and in Uganda, where both urban and rural prevalence were higher in the population-based survey than those derived from antenatal clinic surveillance. In both Chad and Equatorial Guinea, antenatal clinic surveillance systems are generally weak and the projections were based on limited data. Prevalence ratios in countries with a higher adult prevalence, notably in southern and East Africa, were in general closer to 1 than in countries in West and Central Africa and in the Caribbean with a lower national prevalence. Median prevalence ratios (population-based survey: antenatal clinic) are shown for the four regions in figure 1, with a median ratio of 0.9 for both urban and rural areas in southern Africa, 0.94 for urban and 0.89 for rural areas in East Africa, 0.73 for urban and 0.59 for rural areas in West and Central Africa, and 0.45 for urban and 0.87 for rural areas in the Caribbean. The countries included in the regional analysis, however, might not be entirely representative of the respective regions, particularly in southern Africa where some countries (Botswana, South Africa and Swaziland) were excluded from the analysis because of the lack of urban and rural specific data.

**Figure 2:**
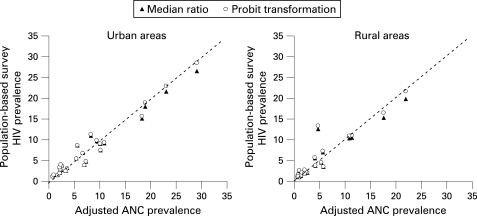
Adjusted antenatal clinic-derived prevalence using either probit transformations or the median prevalence ratio (survey: antenatal clinic), plotted against the “gold standard” population-based survey prevalence for 26 countries with generalised epidemics.

Applying correction factors to antenatal clinic-derived prevalence using either the probit-transformed median difference or the untransformed median prevalence ratios produced adjusted prevalence estimates that agreed well with the “gold standard” population-based survey prevalences in both urban and rural areas (table 2 and fig 2), with slightly better agreement using the probit transformed median difference. A notable exception was in rural areas of the Central African Republic where the survey prevalence was 4.7%, the antenatal clinic-derived prevalence was 15.4% and the adjusted antenatal clinic-based estimates using the two correction factors were around 13%. Antenatal clinic surveillance data for Central African Republic are sparse and 2002 was the last year for which surveillance data were available. More recent data are therefore required from antenatal clinics to obtain more accurate estimates of HIV prevalence over time.

**Table 2:** Antenatal clinic (ANC) surveillance-based estimates of HIV prevalence adjusted using either the median of the survey to antenatal clinic-based prevalence ratios, or the median of the probit-transformed prevalence difference; compared to the “gold standard” population-based survey prevalence

Country	Urban			Rural		
	National survey adult HIV prev	Adjusted ANC prevalence using median ratio	Adjusted ANC prevalence using probit transforms	National survey adult HIV prevalence	Adjusted ANC prevalence using median ratio	Adjusted ANC prevalence using probit transforms
Lesotho	29.1	26.6	28.5	21.9	19.8	21.5
Malawi	18.3	15.2	15.7	11.3	10.4	11.0
Zambia	23.1	21.7	22.9	10.8	10.3	10.9
Zimbabwe	18.9	18.0	18.9	17.6	15.3	16.4
Burundi	9.4	9.7	9.8	2.5	2.0	2.0
Ethiopia	5.6	8.6	8.7	0.7	1.6	1.5
Kenya	10.0	9.1	9.1	5.6	7.0	7.2
Rwanda	7.3	4.9	4.7	2.2	2.2	2.2
Tanzania	10.9	9.2	9.3	5.3	4.5	4.6
Uganda	10.1	7.6	7.6	5.7	3.5	3.5
Benin	1.7	1.6	1.5	0.9	1.0	0.9
Burkina Faso	3.6	3.2	3.1	1.3	1.3	1.3
Cameroon	6.7	6.8	6.8	4.0	5.5	5.7
Central African Republic	8.3	11.0	11.2	4.7	12.5	13.3
Chad	7.0	4.0	3.9	2.3	2.3	2.3
Cote d’Ivoire	5.4	5.5	5.4	4.1	3.7	3.8
Equatorial Guinea	3.3	2.6	2.4	3.1		
Ghana	2.3	2.9	2.7	2.0	2.7	2.7
Guinea	2.4	4.1	3.9	1.0	2.6	2.6
Liberia	2.5	4.0	3.8	0.8		
Mali	2.2	3.0	2.8	1.5	2.1	2.1
Niger	2.1	2.1	1.9	0.6	1.1	1.0
Senegal	0.7	1.2	1.1	0.7	1.1	1.1
Sierra Leone	2.1	3.5	3.3	1.3		
Haiti	2.3	4.2	4.1	2.0	1.9	1.8
Dominican Republic	0.9	1.6	1.5	1.2		

## DISCUSSION

Comparison of HIV prevalence data from 26 countries with generalised HIV epidemics show that unadjusted estimates of adult HIV prevalence derived from antenatal clinic surveillance are almost always higher than those from national population-based household surveys.

While antenatal clinic surveillance and population-based surveys both have strengths and weaknesses, the analysis of data from the two sources provides complementary information on both the point prevalence of HIV in countries as well as information on epidemic trends. In particular, antenatal clinic-based surveillance provides good data on epidemic trends over time, while population-based surveys provide geographically more representative estimates of adult HIV prevalence at a particular point in time.

Analysis of population-based survey data in Ethiopia, Kenya, Malawi, Tanzania and Uganda showed that when the population was stratified to match the antenatal clinic population, the HIV prevalence estimates were similar.^29^ Studies also show that non-response is unlikely to have a significant effect on HIV prevalence measured from population-based surveys conducted in countries with generalised epidemics.^9^ ^13^ Only in countries with high levels of non-response or large differences in HIV prevalence between survey responders and non-responders is the overall prevalence expected to be affected and in these situations prevalence should be adjusted for prevalence among non-responders.^9^ ^12^

Using national population-based surveys as “gold standard” for estimating adult prevalence, the results of this analysis show that prevalence derived from antenatal clinic data have to be adjusted downwards by about 20% (95% CI 10% to 30%) in both urban and rural areas to obtain more accurate estimates of national adult HIV prevalence. Applying the median difference between probit-transformed survey and antenatal clinic-based prevalence estimates to antenatal clinic-based prevalence in each country resulted in predicted “gold standard” estimates that were slightly more accurate than using the untransformed median ratio of survey to antenatal clinic-based prevalence. Countries in southern and East Africa where national prevalence is higher appear to require a slightly smaller adjustment than those countries in West and Central Africa and in the Caribbean with a relatively lower national prevalence.

Since 2001, UNAIDS has been adjusting antenatal clinic-based prevalence estimates in rural areas, but not in urban areas, downward by about 20% to account for the exclusion of sites in remote areas from surveillance efforts.^5^ However, the analysis and correction factors derived in this paper indicate that prevalence in both rural and urban areas should be adjusted downwards by about 20%.

The analysis presented in this paper has been incorporated in the UNAIDS estimates of HIV prevalence and impact in countries with generalised epidemics for 2007, following recommendations of the UNAIDS Reference Group on Estimates, Modelling and Projections.^30^ Using the latest version of EPP,^17^ antenatal clinic-based HIV prevalence curves were adjusted as follows: prevalence curves for countries in which national population-based surveys have been conducted were calibrated to match the survey estimate for the year in which the survey was conducted. For countries in which national population-based surveys have not been conducted, antenatal clinic-based prevalence curves in both urban and rural areas were adjusted downwards on average by about 20%, according to the probit-transformed correction factor described in this paper. Adult HIV prevalence in sub-Saharan Africa in 2005 was estimated at 6.1%.^6^ The revised estimate for 2005 in this region as estimated in 2007, using additional surveillance data and revised methods, was 5.4% (epidemiology slides available on www.unaids.org). In addition to having improved surveillance data, the downward revision in estimates can be partly explained by calibration of sentinel surveillance in a few additional countries that have conducted household surveys during this period (including Benin, Central African Republic, Liberia, Malawi, Mali and Swaziland), as well as by the additional downward adjustment of prevalence in urban areas in countries where household surveys have not been conducted.

A potential limitation of the analysis presented in this paper relates to the quality of antenatal clinic surveillance in countries. In the early stages of the epidemic, surveillance often includes settings where there are known cases of HIV infection and the analysis of data from these settings might lead to an overestimation of the national prevalence. As surveillance systems expand, more sentinel sites are included, often from areas with lower prevalence than those initially included, and prevalence might be reduced. In many countries included in this analysis, including all countries in southern and East Africa, the quality of surveillance is regarded as good, as shown elsewhere in this issue.^31^ However, in some countries in West and Central Africa surveillance systems are only partially functioning and expansion or improvement of the surveillance systems might affect estimated prevalence and the correction factor needed to adjust antenatal clinic-based prevalence. Furthermore, expansion of treatment and other services might in future affect antenatal clinic attendance patterns and estimated prevalence, which could also affect the correction factor. It is therefore recommended that the analysis presented in this paper be updated on a regular basis as more data become available to ensure the best possible estimation of the epidemic and its impact.

### Conclusion

A comparison of adult HIV prevalence obtained from national population-based surveys and antenatal clinic surveillance shows that unadjusted estimates derived from antenatal clinic surveillance generally overestimate national adult HIV prevalence in countries with generalised epidemics. Providing that the methodology used in national population-based surveys is sound and reliable, and potential bias of survey non-response is adjusted for, the population-based survey estimates can be used to adjust antenatal clinic-based prevalence in countries where such surveys have been conducted. In countries where population-based surveys have not been conducted, the recommendation, based on the analysis in this paper, is that estimates of prevalence based on antenatal clinic surveillance in both urban and rural areas should be adjusted downward by about 20%.
